# Quercetin is a foe in the heart by targeting the hERG potassium channel

**DOI:** 10.22038/ijbms.2024.77846.16848

**Published:** 2024

**Authors:** Zihao Lu, Shuwen Li, Rui Wei, Wenwen Li, Yuqian Huang, Tingting Yang, Meng Yan

**Affiliations:** 1 Jiangsu Key Laboratory of New Drug Research and Clinical Pharmacy, Xuzhou Medical University, Xuzhou, China; 2 Department of Pharmacy, Jiangsu Province Official Hospital, China; # These authors contributed equally to this work

**Keywords:** Degradation, KCNH2, Long QT syndrome, Nedd4, Ubiquitination

## Abstract

**Objective(s):**

Quercetin is a plant flavonoid known for its pharmacological activities, such as antioxidant, anti-inflammatory, and anti-cancer properties. However, there is limited information available regarding its potential toxicities. A previous study showed that quercetin can inhibit human ether-a-go-related gene (hERG, also named KCNH2) currents, which may lead to long QT syndrome, torsade de pointes (TdP), and even sudden cardiac death. This study aimed to investigate the effects of quercetin on hERG and its potential mechanism.

**Materials and Methods:**

hERG currents and action potential duration (APD) were assessed using the patch clamp technique. Molecular docking was employed to elucidate the binding sites between quercetin and hERG. Transfection of wild-type or mutant plasmids was used to verify the results of molecular docking. Western blot was performed to determine the expression levels of hERG, transcription factor SP1, molecular chaperones HSP70 and HSP90, phosphorylated E3 ubiquitin ligase p-Nedd4-2, serum- and glucocorticoid-inducible kinase (SGK1), and phosphatidylinositol 3-kinase (PI3K). Immunoprecipitation was conducted to evaluate hERG ubiquitination.

**Results:**

Quercetin acutely blocked hERG current by binding to F656 amino acid residue, subsequently accelerating channel inactivation. Long-term incubation of quercetin accelerates Nedd4-2-mediated ubiquitination degradation of hERG channels by inhibiting the PI3K/SGK1 signaling pathway. Moreover, the APD of human induced pluripotent stem cell-derived cardiomyocytes (hiPS-CMs) is significantly prolonged by 30 μM quercetin.

**Conclusion:**

Quercetin has a potential risk of proarrhythmia, which provided useful information for the usage and development of quercetin as a medication.

## Introduction

Acquired long QT syndrome (acLQTS) is a life-threatening cardiac arrhythmia characterized by QT interval prolongation which may increase the risk for torsades des pointes (TdP) ventricular tachycardia and sudden cardiac death (SCD)(1). Currently, drug-triggered acLQTS accounts for over 50% of this disease. The iconic mechanism of acLQTS is the inhibition of human ether a go-related gene (hERG) potassium channels, conferring rapid activating component of delayed rectifier potassium channel current (I_kr_) which is crucial for the third stage repolarization of cardiac action potentials (2). In the last century, many drugs were withdrawn from the market due to the serious adverse cardiac reactions associated with hERG blockage (3). Therefore, it is necessary to test the effect of new drugs on hERG channels. 

Quercetin is a well-known natural flavonoid and exists widely in all kinds of fruits and vegetables. Modern pharmacological studies have demonstrated that quercetin is involved in extensive biological activities including anti-oxidation, anti-aging, anti-inflammatory, etc. Nevertheless, few studies have focused on the toxic effects of quercetin. Continuously growing evidence indicates that flavonoids may interact with the hERG channel and block it (4). Therefore, quercetin may have an inhibitory effect on the hERG channel, posing a risk of developing Tdp or even SCD. The mechanisms of drug-induced hERG channel deficiency mainly have two types: (1) acutely blocking the hERG channel; and (2) inhibiting channel protein expression via disrupting channel protein synthesis, trafficking, or degradation after long-term incubation. Previous studies reported that quercetin acutely blocks the hERG channel (5). However, the underlying mechanisms and the long-term effects of quercetin on the hERG channel have not been elucidated.

In this study, our results demonstrated that quercetin acutely blocks the hERG channel and accelerates its inactivation, partially mediated by the interaction with F656. Long-term incubation of quercetin accelerates Nedd4-2-mediated ubiquitination degradation of hERG channels by inhibiting the PI3K/SGK1 signaling pathway. Furthermore, we explored the effect of quercetin on the APD of hiPS-CMs. Our study focused on the adverse cardiac reactions that should be noted after introducing quercetin into the pharmaceutical market, especially in patients with potential cardiac diseases.

## Materials and Methods

***Cell culture***

HEK293 cells stably expressing the wild-type hERG gene (hERG-HEK293), purchased from Hunan Fenghui Biotechnology Co., Ltd, were cultured at 37 ^°^C and 5% CO_2 _in Dulbecco’s Modified Eagle’s Medium (DMEM, Bio-channel, China) supplemented with 10% fetal bovine serum (FBS, Sunrise, United States) and 1% penicillin-streptomycin solution (Bio-channel, China). 

***Molecular docking***

A molecular docking study was performed to investigate the binding mode between quercetin and hERG using Autodock vina 1.1.2 (6). The three-dimensional (3D) structure of the hERG (PDB ID: 5VA1) was downloaded from the RCSB Protein Data Bank (http://www.rcsb.org/pdb/home/home.do). The 2D structure of the compound was drawn by ChemBioDraw Ultra 14.0 and converted to the 3D structure by ChemBio3D Ultra 14.0 software. The AutoDockTools 1.5.6 package (7, 8) was employed to generate the docking input files. The ligand was prepared for docking by merging non-polar hydrogen atoms and defining rotatable bonds. The search grid of the hERG site was identified as center_x: 77.387, center_y: 62.935, and center_z: 81.695 with dimensions: size_x: 16, size_y: 16, and size_z: 16. In order to increase the docking accuracy, the value of exhaustiveness was set to 20. For Vina docking, the default parameters were used if not mentioned. The best-scoring pose as judged by the Vina docking score was chosen and visually analyzed using PyMoL 1.7.6 software (http://www.pymol.org/).

***Western blot***

RIPA lysate (Beyotime, China) and PMSF (100 mM, Beyotime, China) were used to extract total proteins from hERG-HEK293 cells which were treated with quercetin (3, 10, 30 μM) for 24 hr. The 120 μg total proteins were separated with the SDS-PAGE Gel Quick Preparation Kit and transferred onto nitrocellulose filter (NC) membranes. The NC membranes were closed by 30 ml of PBS containing 0.9 g Albumin Bovine V (Solarbio, China) for 1 hr. Then, the NC membranes were incubated in the same solution containing 1:1000 primary antibodies against β-actin and in the same solution containing 1:200 primary antibodies against hERG at 4 ^°^C overnight. The NC membranes were washed in PBST 3 times and incubated with 1:1000 dilution of goat anti-mouse IgG (Vicmed, Germany) labeled by horseradish peroxidase for 1 hr. The bands were detected using a two-color infrared laser imaging system, then images were processed by Image J.

***Real-time quantitative RT-PCR***

TRIzol Universal Reagent (Tiangen, China) was used to extract total RNA from hERG-HEK293 cells, and the quantity of total RNA was detected using a spectrophotometer (Thermo, 00113711, USA). cDNA was reversely transcribed with 2 μg of total RNA using the ReverTra Ace qPCR RT Kit (TOYOBO, FSQ-101, Japan) in a 2720 Thermal Cycler. The primers for GAPDF and hERG were purchased from Shanghai Shenggong. Real-time PCR analysis was performed by Real-time fluorescence quantitative PCR instrument.

***Immunofluorescence***

hERG-HEK293 cells in a glass petri dish were washed thrice with cold PBS and fixed at -20 ^°^C for 20 min with cold methanol. They were washed in PBS three times and then blocked at room temperature with 2% BSA for 1 hr. The sections were incubated with a primary antibody against hERG at 4 ^°^C overnight, and then incubated with a secondary antibody conjugated with DyLight 488 at 37 ^°^C for 1 hr. Then, the sections were stained with DAPI and were viewed with an Olympus BX43F fluorescence microscope (Tokyo, Japan).

***Immunoprecipitation***

The hERG cells were transferred to a 1.5 ml tube after washing with PBS and then centrifuged at 3,000 rpm for 5 min. Total proteins were harvested in lysis buffer and determined by NanoDrop One (Thermo Fisher Scientific). Anti-hERG, or anti-Ub antibody was separately mixed with the protein, and the mixture was placed on a 360^°^ shaker overnight at 4 ^°^C. Subsequently, beads (Santa Cruz A-G SC-2003) were added to the mixture and then placed on a shaker overnight at 4 ^°^C. The mixture was centrifuged for 5 min at 1,500 rpm, and the pellet was washed with PBST 6 times. Next, the loading buffer was added to the pellet, and the tubes were boiled for 10 min, followed by centrifugation. The supernatants were collected and separated via SDS-PAGE and transferred to the NC membrane, blocked with 3% BSA, and incubated with antibodies against hERG, or Ub at 4 ^°^C overnight. After the membranes were incubated with secondary antibodies for 1 h at room temperature, the blots were analyzed through western blot analysis.

***Transfection***

The hERG mutations F656A (phenylalanine to alanine) cDNA were obtained from The Addgene Repository (http://www.addgene.org)(9). WT or F656A cDNA were transfected into HEK293 cells. In brief, cells were plated in 25-mm dishes at the required density and incubated overnight. 3-4 hr prior to transfection, the media on the dishes was changed to 4.5 ml DMEM. Next, 250 µl opti-MEM and 12.5 µl Lipofectamine 2000 (Invitrogen) were added to a tube labeled A, 250 µl opti-DMEM and 2 µg siRNA or 4 µg cDNA were added to a tube labeled B, then mixed solution A and solution B. After incubation at room temperature for 20 min, the mixture was added to cells in the dishes and the cells incubated at 37 ^°^C in a humidified CO_2_ incubator. The transfection was 48 hr prior to analysis.

***Patch clamp techniques***

Currents and APD were measured using the whole-cell patch clamp technique under voltage-clamp and current-clamp mode, respectively. The procedures were described previously (2). Axopatch-200B patch-clamp amplifier and Clampex 10.2 (Axon Instruments, USA) were used to record the traces, generate protocols, and acquire data. Patch pipettes with resistances of 1-3 MΩ when filled with the corresponding pipette solution were used to perform whole-cell configuration. Cells were treated with drug solution overnight before measurement.

hERG currents were recorded from hERG-HEK293 cells. The extracellular solution was Ca^2+^-containing Tyrode’s solution (136 mM NaCl, 5.4 mM KCl, 10 mM HEPES, 1 mM MgCl_2_·6H_2_O, 1.8 mM CaCl_2_, and 10 mM glucose, with pH 7.4). The pipette solution contained 130 mM KCl, 1 mM MgCl_2_·6H_2_O, 10 mM HEPES, 5 mM Mg-ATP, 5 mM EGTA, and 0.1 mM GTP (pH 7.3 with KOH).

Action potentials were measured from hiPS-CMs. The extracellular solution was Ca^2+^-containing Tyrode’s solution. The pipette solution for APD recorded in hiPS-CMs contained 120 mM KCl, 1 mM MgCl_2_·6H_2_O, 10 mM HEPES, 3 mM Mg-ATP, and 10 mM EGTA (pH 7.3 with KOH). 

***Agents and antibodies***

Quercetin (≥95% purity, Q4951–100G) was obtained from Sigma (MA, USA). Primary antibodies for hERG (SC-377388) and Ub (SC-8017) were purchased from Santa Cruz Biotechnology (Santa Cruz, USA). Antibodies against SGK1 (28454-1-AP) were purchased from Proteintech (Wuhan, China). Antibodies against p-Nedd4-2 were purchased from Cell Signalling Technology (Boston, USA), and antibodies against PI3K (WL02240) were purchased from Wanleibio (Shenyang, China). Antibodies against HSP70 (M20041), and HSP90 (M20032F) were from Abmart (Shanghai, China). β-actin antibody was purchased from Bioworld Technology (St. Louis, USA). IRDye 800CW goat anti-mouse IgG (V926-32210) and IRDye 800CW goat anti-rabbit IgG (V926-32211) were purchased from Vicmed (Xuzhou, China). RIPA lysis buffer and PMSF lysis buffer were purchased from Beyotime (Nanjing, China). Alexa fluor 488 goat anti-mouse IgG (A11029) was purchased from Thermo Fisher Scientific (MA, USA). Protein A/G PLUS-Agarose (SC-2003) was from Santa Cruz Biotechnology (Santa Cruz, USA).

***Statistical analysis***

Data are presented as means ± standard error of the mean (SEM). ANOVA and Student’s t-test were used to identify the differences between the means. *P*-values<0.05 were considered statistically significant. GraphPad Prism 8.0 and Origin 9.0 were used for statistical analysis and graphing.

## Results

***Quercetin acutely blocks the hERG channel***

**Figure 1:**
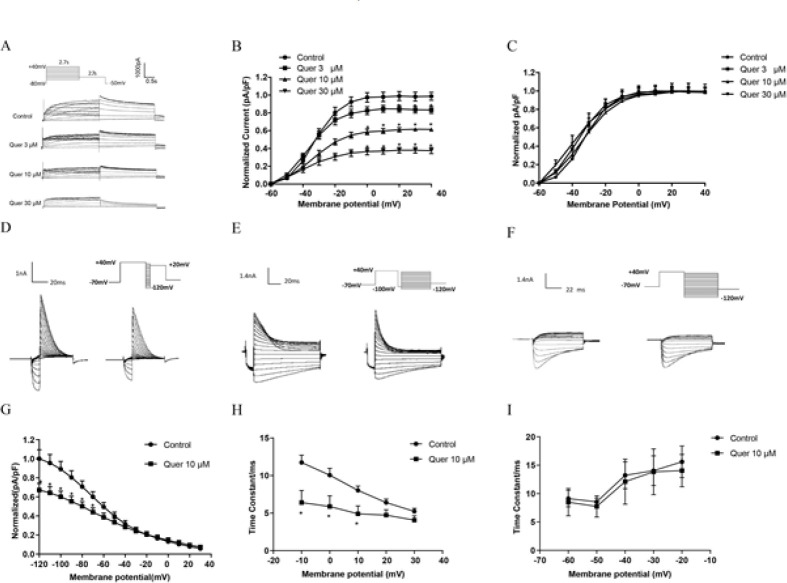
Quercetin acutely blocks the hERG channel

The hERG currents were recorded under controlled conditions, and then different concentrations of quercetin were washed into the bath for 10 min at room temperature (Figure 1A). The peak hERG tail currents were measured, normalized for each cell to the control value, and then averaged at drug concentrations of 3 μM, 10 μM, and 30 μM (Figure 1B). The efficiency of inhibition was computed from the tail current at +40 mV with the function: (*I*_Drug_ - *I*_Control_)· *I*_Control_^-1^. In the presence of quercetin, hERG current density was decreased by 15.62±3.92% (3 μM), 37.70±2.35% (10 μM), and 62.10±3.35*% *(30 μM) relative to the control group. Next, we detected the effect of quercetin on hERG channel dynamics. Current tracing for steady-state inactivation, onset of inactivation, and recovery were recorded (Figure 1D-F). Activation and steady-state inactivation curves were shown in Figures 1C and 1G, and the time constant of the inactivation and recovery from the onset of inactivation were present in Figures 1H and 1I. From the figures, we can see that quercetin accelerated the inactivation of hERG channels and shortened the inactivation time constant. The time constant was shortened from 11.74±0.97 ms to 6.40±1.62 ms at +20 mV. It means that the hERG channel closed more quickly in the presence of 10 μM quercetin. However, quercetin showed no significant effect on the activation and recovery. Taken together, quercetin acutely inhibited hERG channels, which is at least partially attributed to the changes in hERG channel dynamics.

***Phe656 binding accounts for quercetin-induced acute inhibition of the hERG channel***

**Figure 2:**
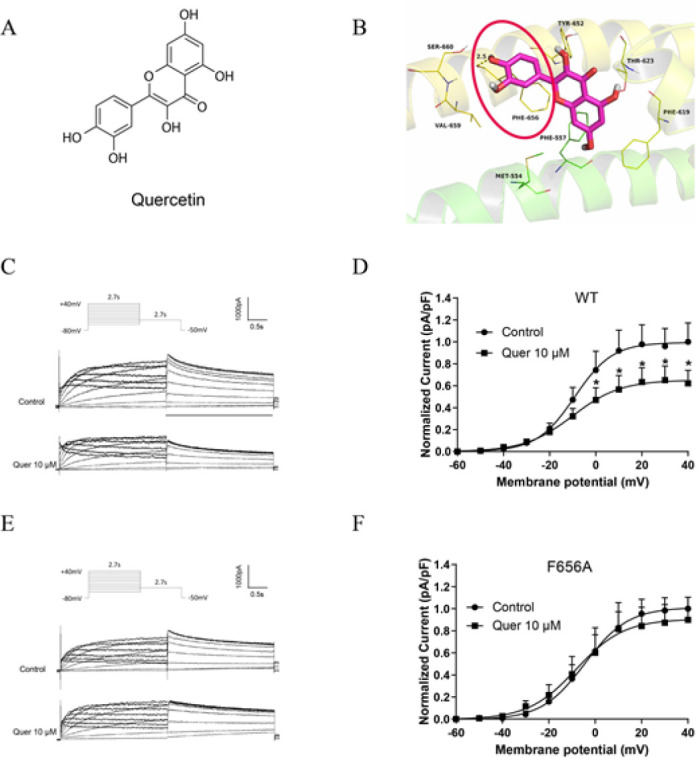
Quercetin trapped in human ether a go-related gene (hERG) channels and docked to the amino residues

To further explore the molecular interaction between quercetin and the hERG channel, molecular docking was performed by computer modeling. The chemical structure of quercetin and molecular docking results are shown in Figure 2A-B. As presented in Figure 2B, quercetin adopts a compact conformation to bind at the surface of the hERG channel and is surrounded by several amino residues to form a strong hydrophobic binding. Detailed analysis showed that the phenyl group of the quercetin formed π-π stacking interaction with the residue Phe-656. Moreover, one key hydrogen bond interaction was observed between the quercetin and the residue Phe-656, with a bond length of 2.5 Å, which was the main interaction between the quercetin and the hERG. 

To clarify whether hERG channel acute blockage caused by quercetin was on account of Phe656 binding, we studied the effects of quercetin on mutant channels by transfecting HEK293 cells with WT-hERG or F656V-hERG. The electrophysiological results showed that quercetin (10 μM) significantly inhibited WT-hERG tail current with an inhibition ratio of 37.7% under +40 mV, while quercetin (10 μM) did not affect F656V-hERG tail current (Figure 2C–F). These results suggested that Phe656 binding accounts for quercetin-induced acute inhibition of the hERG channel.

***Quercetin inhibits hERG channel expression after long-term incubation***

**Figure 3:**
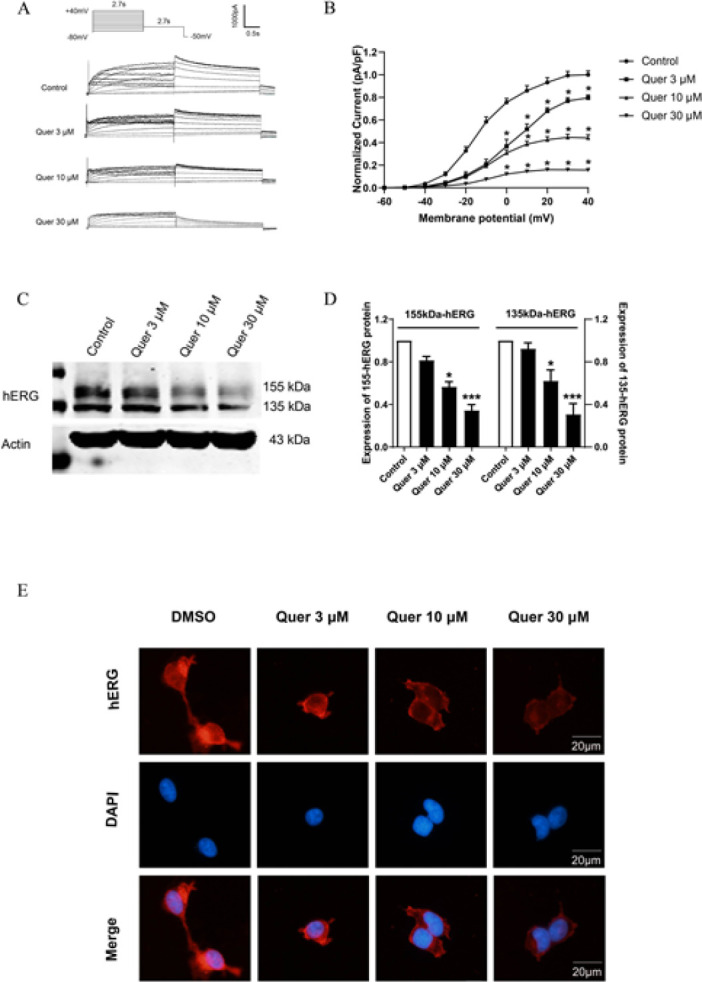
Long-term effect of quercetin on hERG channels

Next, we determined the long-term effect of quercetin on hERG channels, hERG-HEK293 cells were treated with different concentrations of quercetin (3, 10, and 30 μM) for 24 hr. hERG currents were recorded by the patch clamp technique. As shown in Figure 3A-B, quercetin incubation reduced hERG tail current by 20.5%±2.6% (3 μM), 55.8%±2.6% (10 μM), and 84.2%±0.6% (30 μM). We also examined hERG protein expression using western blotting. Consistent with the electrophysiological results, quercetin caused a prominent decrease in hERG protein levels. Mature 155 kDa hERG and immature 135 kDa hERG were reduced to 18.5%±2.6% (3 μM), 43.5%±3.4% (10 μM), 65.6%±3.9% (30 μM), 7.7%±3.9% (3 μM), 37.8%±7.2% (10 μM), and 69.3%±7.1% (30 μM), respectively (Figure 3C-D). Immunofluorescence analyses also showed that quercetin remarkably reduced hERG protein expression (Figure 3E). These findings implicated that quercetin incubation inhibited hERG channel expression in a concentration-dependent manner.

***Quercetin accelerates the degradation of the hERG channel on the cell surface***

**Figure 4:**
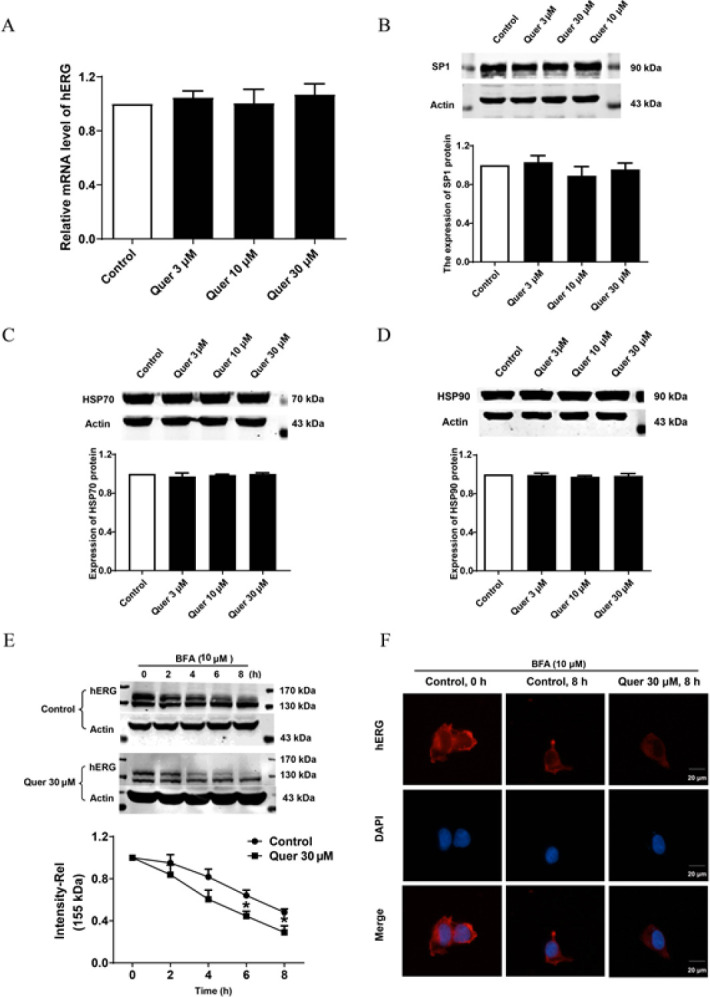
Quercetin accelerates the degradation of the hERG channel on the cell surface

We first examined the effect of quercetin on the mRNA expression level of hERG, the results showed that quercetin (3, 10, and 30 μM) has no significant effect on hERG mRNA (Figure 4A). Simultaneous results that quercetin did not alter the expression of transcription factor SP1 which is responsible for hERG channel transcription were displayed in Figure 4B. These results indicated that quercetin does not affect hERG transcription. Next, we detect the expression of chaperone HSP70 and HSP90 in the presence of quercetin. Surprisingly, quercetin did not affect the two molecules crucial for hERG channel trafficking from ER to Golgi (Figure 4C-D). Then, we studied the effect of quercetin on hERG degradation in which brefeldin A (BFA, 10 µM) was used to block protein trafficking from ER to Golgi. Figure 4E shows time-dependent degradation of the 155-kDa hERG protein in control and quercetin (30 μM) treated cells after BFA treatments. Compared with the control group, quercetin treatment markedly accelerated the degradation rate of hERG, reflected in that the 155-kDa hERG band intensity decreased by 53.1±2.6% in control cells; whereas it decreased by 68.4±5.3% in quercetin-treated cells after an 8 hr BFA-incubation. Consistent with these findings, immunofluorescence results (Figure 4F) showed that following BFA incubation, quercetin significantly reduced mature hERG protein (red on cell membrane). Together, quercetin accelerates the degradation of mature hERG channels.

***Quercetin promotes hERG protein degradation by inhibiting the PI3K/SGK1 pathway***

**Figure 5:**
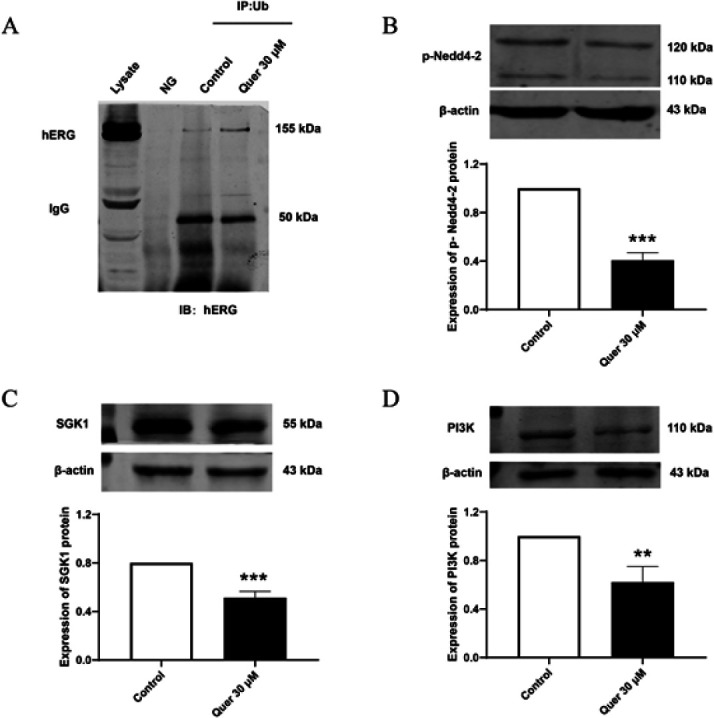
Quercetin promotes hERG protein degradation by inhibiting the PI3K/SGK1 pathway

To explore the mechanism underlying quercetin-triggered hERG channel degradation, we analyzed the influence of quercetin (30 μM) on hERG ubiquitination. Co-immunoprecipitation results showed that quercetin could significantly promote hERG ubiquitination (Figure 5A). Considering that the hERG channel is a direct substrate of Nedd4-2, we tested the expression level of p-Nedd4-2 and the upstream kinase SGK1. Western blot results showed that the phosphorylation of Nedd4-2 decreased in the presence of 30 μM quercetin, along with the inhibition of SGK1 (Figure 5B-C). We also analyzed the expression of PI3K, the upstream effector of SGK1, and the protein expression level was significantly down-regulated after quercetin treatment (Figure 5D). These results suggested that quercetin promotes Nedd4-2-mediated hERG ubiquitination degradation by inhibiting the PI3K signaling.

***Quercetin prolongs APD in hiPS-CMs***

**Figure 6:**
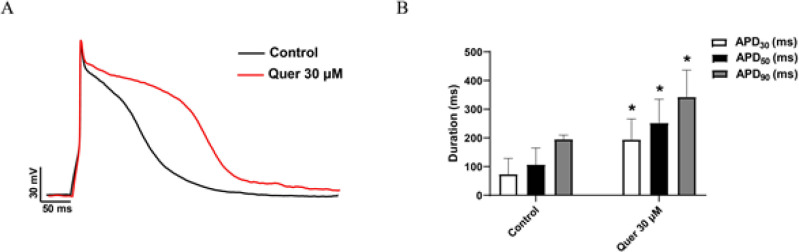
Effects of quercetin on the APD of hiPS-CMs

To test whether quercetin could further change APD due to hERG channel deficiency, we performed patch-clamp experiments in hiPS-CMs. Figure 6 shows that APD in hiPS-CMs is significantly prolonged by 30 μM quercetin. APD_30_ was prolonged from 73.42±55.29 ms to 193.88±72.35 ms, APD_50_ was prolonged from 106.73±58.18 ms to 252.42±82.39 ms, and APD_90_ was prolonged from 195.14±14.64 ms to 342.86±93.70 ms (Table 1). These results indicate that quercetin has the potential to induce LQTS.

## Discussion

In the present studies, we demonstrated that (1) quercetin acutely inhibited hERG currents partially due to the acceleration of channel deactivation, (2) residue Phe656, an essential component of hERG binding sites, mediated hERG current inhibition by quercetin, (3) quercetin promoted Nedd4-2 mediated hERG ubiquitination degradation via inhibiting PI3K/SGK1 pathway, and (4) quercetin prolonged APD of hiPS-CMs.

Quercetin is an organic flavonoid present in several fruits and vegetables with various pharmacological properties which were demonstrated by this dietary supplement as a possible treatment for inflammatory diseases and cancer. Unfortunately, conflicting research has cast uncertainties on the toxicity of quercetin (10, 11). Numerous flavonoids exhibited direct and/or indirect inhibition of the hERG channel current (12, 13). Sun *et al*. discovered that quercetin is a potent hERG channel inhibitor (5). However, there is a lack of more in-depth research. In our study, we comprehensively examined the effects of quercetin on the hERG channel.

hERG potassium channel is crucial for phase 3 rapid repolarization of the cardiac action potential. A large variety of medications trigger LQTS by targeting hERG channels (14, 15). The mechanism of drug-induced hERG channel deficiency possesses two types: (1) directly blocking the hERG channel; and (2) indirectly disrupting channel protein synthesis, trafficking, or degradation (16). In our research, we first tested the inhibitory effect of different concentrations of quercetin on hERG currents after a 10-minute bath. Ten μM quercetin decreased hERG current density by 37.70 ± 2.35% (Figure 1A-B), which is consistent with previous results (5). Next, hERG channel dynamics results showed that quercetin accelerated the inactivation of hERG channels and shortened the inactivation time constant without affecting the activation and recovery (Figure 1D-F). The direct mechanism underlying drug-induced hERG blockage proposed is binding to the amino acid residues (Tyr652 and Phe656) in the S6 transmembrane helix of the hERG channel subunit (17). High-affinity dofetilide binding to hERG expressed in Xenopus Oocytes involves Phe656 residue (18). Class IA antiarrhythmic drug quinidine blocks the hERG channel influenced by Tyr652 residue (19). Our molecular docking results displayed that quercetin formed π-π stacking and one key hydrogen bond interaction with the residue Phe-656 (Figure 2B). To verify our analysis, we recorded WT-hERG or F656V-hERG currents in the presence of quercetin, and the results suggested that Phe656 binding accounts for quercetin-induced acute inhibition of the hERG channel (Figure 2C-F).

The great majority of drugs reduce hERG currents by inhibiting the expression of channel protein (20-24). In the process of hERG biosynthesis, the hERG gene is first translated and initially glycosylated in ER to form the immature channel with a molecular weight of 135 kDa. With the assistance of chaperones, it is then appropriately folded and transported to Golgi for secondary glycosylation to form a mature channel with a molecular weight of 155 kDa. Finally, it is transported to the cell membrane to perform its physiological functions (25). In the present research, we found that quercetin incubation inhibited hERG protein expression and reduced channel currents (Figure 3). Mechanistically, we investigated the effect of quercetin on hERG channel protein expression from three aspects: transcriptional level, post-transcriptional transport, and cell membrane protein degradation. At the transcription level, quercetin did not alter the mRNA level of the hERG channel, as well as the protein expression of transcription factor SP1 (Figure 4A-B), which is essential to driving the hERG promoter thereby transcription (26). The most common mechanism for drugs to inhibit hERG protein expression is trafficking defect (2, 27). So, we detected the expression of chaperone HSP70 and HSP90 in the presence of quercetin. Surprisingly, quercetin did not affect the two molecules that are crucial for hERG channel trafficking from ER to Golgi (Figure 4C-D). Some drugs down-regulated hERG expression by increasing membrane channel protein degradation. Arsenic trioxide and probucol were reported to accelerate hERG protein degradation from the cell membrane (2, 23). Our study also suggested that quercetin accelerates the degradation of mature hERG channels (Figure 4E-F).

Research has shown that tyrosine kinases may participate in the post-transcriptional regulation of cardiac ion channels (28). Moreover, tyrosine kinase inhibitors and the downstream PI3K inhibitors in recent years have received a growing concern of their cardiotoxicity which is linked to increased blockage of hERG channels (29, 30). Prevention of PI3K activation by pharmacological inhibition or molecular inactivation significantly suppressed hERG function (31). While, quercetin is an inhibitor of tyrosine kinase, which can inhibit the phosphorylation activity of downstream PI3K in many cell types (28, 32). Thus, quercetin may down-regulate hERG expression via PI3K-mediated post-transcriptional regulation. A recent study showed that tyrosine kinase inhibitors nilotinib and vandetanib decreased hERG membrane protein expression, thus increasing the incidence of arrhythmia in hiPS-CMs. SGK1 is a downstream effector of PI3K (33, 34). Cui *et al*. found that an SGK1 activator, C4-ceramide, prevented those two drugs-induced hERG protein down-regulation (28). Previous research showed that the hERG channel is a direct substrate of the E3 ubiquitin ligase Nedd4-2, which is the downstream target of SGK1 (35, 36). Consequently, we hypothesized that quercetin inhibited hERG channel expression by promoting Nedd4-2-mediated degradation via the PI3K/SGK1 pathway. In our study, quercetin showed an inhibitory effect on PI3K expression. Consistently, we found that the phosphorylation of Nedd4-2 decreased in the presence of quercetin, along with the inhibition of SGK1 (Figure 5B-D).

Mature hiPS-CM exhibits similar characteristics to adult cardiomyocytes in many aspects (37). Due to its ease of operation and accuracy in results, *in vitro* experiments of hiPS-CMs are gradually replacing *in vivo *and* in vitro* animal experiments (2, 38). Many investigators have successfully utilized mature hiPS-CMs for the research of cardiac diseases, drug cardiotoxicity screening, and cardiac medication efficacy testing (39, 40). In our study, we explore the potential effect of quercetin on the APD of hiPS-CMs. We demonstrated that quercetin markedly prolonged the APD of hiPS-CMs (Figure 6 and Table 1), suggesting the pro-arrhythmic potential and toxicity of quercetin on the heart. Some scholars argue that quercetin has a low probability of inducing cardiotoxicity by hERG blocked at a clinically relevant concentration (41). In a randomized, single-blind study, the maximum plasma concentration (*C*_max_) of quercetin is 1.14±0.22 μM in healthy subjects administered a single oral dose of 163 mg quercetin (42). It seems to be far below the 50% inhibiting concentration (*IC*_50_) of hERG (approximately 11 μM (5)). However, the recommended daily intake dose of quercetin as a dietary supplement is usually 500-1000 mg d^-1^ (43). Moreover, quercetin has the potential to enhance the nephrotoxicity of damaged kidneys in animal studies involving oral quercetin application (44). Taken together, we believe that long-term use of quercetin as a dietary supplement for individuals with cardiovascular/renal diseases, especially those who take excessive medication, still has a risk of developing heart rate abnormalities due to hERG inhibition.

There are some shortcomings of our paper. On the one hand, it’s not clear whether other mechanisms promote hERG channel degradation in the presence of quercetin. On the other hand, the effect of quercetin on other ion channels (such as the L-type calcium channel and sodium channel, which are important to the action potential of cardiomyocytes) needs to be developed in the future.

(A) Voltage clamp protocol and representative hERG currents recorded from hERG-HEK293 cells under control or quercetin-treated groups. (B) Normalized I-V curve of the tail current in different concentrations of quercetin. n=10. (C) Voltage-dependent activation curves for the control group and groups following exposure to 3 μM, 10 μM, and 30 μM quercetin. Curves were the best fit of the data to a Boltzmann function. n=8. (D) Voltage clamp protocol and representative current tracing for steady-state inactivation. (E) Normalized steady-state inactivation curves. n=8. (F) Voltage clamp protocol and representative current tracing for the onset of inactivation. (G) Time constant of inactivation curves. n=8. (H) Voltage clamp protocol and representative current tracing for recovery. (I) Time constant of recovery curves. n=8. Data are presented as means±SEM. *P<0.05 vs the Control group

hERG: human ether a go-related gene

(A) Chemical structure of Quercetin. (B) Docking diagram of quercetin and hERG. (C, D) Representative currents and normalized I-V curves of WT-hERG current under control condition or 10 μM quercetin treatment. n=10. (E, F) Representative currents and normalized I-V curves of F656A-hERG current under control condition or 10 μM quercetin treatment. n=10. Data are presented as means±SEM. *P<0.05 vs Control group

(A) Voltage clamp protocol and representative hERG currents under control conditions or 3 μM, 10 μM, and 30 μM quercetin treatment for 24 hr. (B) Normalized I-V curve of the tail current in different concentrations of quercetin. n=10. Data are presented as means±SEM. *P<0.05 vs Control group. (C, D) Western blot bands and statistics for hERG channel expression in hERG-HEK293 cells under control conditions or 3 μM, 10 μM, and 30 μM quercetin treatment for 24 hr. n=3. (E) Immunofluorescence images showed reduced hERG protein expression by incubation with 3 μM, 10 μM, and 30 μM quercetin. Scale bar, 20 μm

hERG: human ether a go-related gene

(A) Quercetin (30 μM) treatment does not affect the mRNA level of the hERG channel. (B) Protein expression of transcription factor SP1. (C, D) Protein expression of chaperone molecule HSP70 and HSP90. (E) The effects of quercetin (30 μM) on hERG protein expression in the presence of BFA (10 μM) at different time points. Image densities of 155-kDa hERG bands were normalized to their value measured at t=0. n=5. (F) Immunofluorescence images confirmed application of quercetin promotes hERG degradation. Cells were incubated with quercetin (30 μM) and BFA (10 μM) for 8 h. Scale bar, 20 μm. mean±SEM. *P<0.05 vs Control group

hERG: human ether a go-related gene

(A) Quercetin (30 μM) administration increased hERG ubiquitination. (B) Quercetin administration reduced the p-Nedd4-2 expression level. (C) Quercetin inhibited the protein expression of SGK1. (D) Quercetin inhibited the protein expression of PI3K. Data are shown as mean±SEM. n=5. **P<0.01, ***P<0.001 vs the Control group

hERG: human ether a go-related gene. PI3K: phosphatidylinositol 3-kinase. SGK1: serum- and glucocorticoid-inducible kinase

(A) Representative action potential traces under Control (black line) and 30 μM quercetin (red line) treatment for 24 hr. (B) Statistics of APD30, APD50, and APD90. *P<0.05 vs Control group

APD: action potential duration. hiPS-CMs: human induced pluripotent stem cell-derived cardiomyocytes

**Table 1:** Parameters of APD recorded in hiPS-CMs

	Control	Quer 30 μM
APD_30 _(ms)	73.42 ± 55.29	193.88 ± 72.35*
APD_50 _(ms)	106.73 ± 58.18	252.42 ± 82.39*
APD_90 _(ms)	195.14 ± 14.64	342.86 ± 93.70*
N	4	4

Average values were described as mean±SEM, N indicates the number of cardiomyocytes. *P<0.05 vs Control

APD: action potential duration. hiPS-CMs: human induced pluripotent stem cell-derived cardiomyocytes

## Conclusion

Our study comprehensively investigated the effects and mechanisms of quercetin on the hERG channel and suggested the high proarrhythmic potential of quercetin and provided useful information for the utilization and development of quercetin as a medication.
